# Nipah Virus Exposure in Domestic and Peridomestic Animals Living in Human Outbreak Sites, Bangladesh, 2013–2015

**DOI:** 10.3201/eid2902.221379

**Published:** 2023-02

**Authors:** Ausraful Islam, Deborah L. Cannon, Mohammed Ziaur Rahman, Salah Uddin Khan, Jonathan H. Epstein, Peter Daszak, Stephen P. Luby, Joel M. Montgomery, John D. Klena, Emily S. Gurley

**Affiliations:** icddr,b, Dhaka, Bangladesh (A. Islam, M.Z. Rahman, E.S. Gurley);; Centers for Disease Control and Prevention, Atlanta, Georgia, USA (D.L. Cannon, J.M. Montgomery, J.D. Klena);; Public Health Agency of Canada, Ottawa, Ontario, Canada (S.U. Khan);; EcoHealth Alliance, New York, New York, USA (J.H. Epstein, P. Daszak);; Stanford University, Stanford, California, USA (S.P. Luby);; Johns Hopkins Bloomberg School of Public Health, Baltimore, Maryland, USA (E.S. Gurley)

**Keywords:** Nipah virus, NiV, viruses, henipaviruses, zoonoses, spillover, domestic animals, peridomestic animals, Bangladesh, Indian flying fox, Pteropus medius

## Abstract

Spillovers of Nipah virus (NiV) from *Pteropus* bats to humans occurs frequently in Bangladesh, but the risk for spillover into other animals is poorly understood. We detected NiV antibodies in cattle, dogs, and cats from 6 sites where spillover human NiV infection cases occurred during 2013–2015.

Henipaviruses are batborne zoonoses that have caused fatal neurologic and respiratory disease outbreaks in humans, horses, and pigs. In Bangladesh, the Indian flying fox (*Pteropus medius*) is the known natural reservoir for Nipah virus (NiV). NiV causes annual outbreaks in humans in Bangladesh, where the primary mode of spillover is through consumption of date palm sap contaminated by *P*. *medius* bats (*1*); NiV infection is a particular concern for public health because of the high case-fatality ratio and the risk for person-to-person transmission (*2*).

Domestic and peridomestic animals have been important intermediate hosts for zoonotic henipavirus transmission in outbreaks occurring in Australia, Malaysia, and the Philippines (*3*,*4*). One cross-sectional study suggested possible exposure of livestock to henipaviruses in Bangladesh (*5*). Three instances in which animal contact was associated with human NiV infections in Bangladesh have been reported (*1*,*6*,*7*), although little is known about the transmission mechanisms of henipaviruses into livestock and peridomestic animals in Bangladesh. Our study aimed to detect prior NiV infection among livestock and peridomestic animals living in proximity to humans with spillover cases and identify possible exposure pathways in Bangladesh.

## The Study

During January 2013–January 2015, a total of 6 confirmed human Nipah outbreaks were identified through the Nipah surveillance system in Bangladesh (Figure) (*8*). Once an index case-patient was identified, we identified the closest bat roosts to the case-patient’s household and collected urine from underneath the roosts by using plastic tarps. We aliquoted roost urine in cryovials containing lysis buffer, stored them at cryogenic temperatures, and tested them for evidence of NiV RNA. We used extracted RNA from bat roost urine for detecting NiV by using a probe-based real-time reverse transcription PCR assay (*9*). Roosts were located from 150 m to 2 km from the human spillover index case-patient’s household for all 6 outbreaks (Table 1). Ultimately, we sampled only 5 of the 6 roosts; 1 roost could not be sampled because of political unrest. We identified evidence of NiV RNA shedding in urine collected from 4 roosts. We defined a positive sample as one having >1 aliquot with a cycle threshold value <39.

**Figure:**
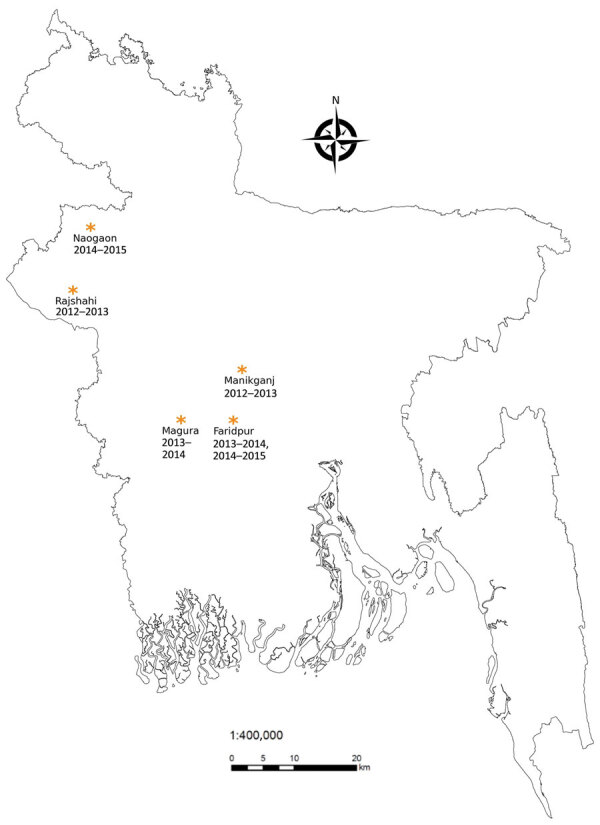
Sites where spillover human Nipah virus infection cases were detected and domestic and peridomestic animals were sampled, Bangladesh, 2013–2015. In Faridpur District, we sampled twice, once during 2013–2014 and once during 2014–2015.

**Table 1:** Spillover human Nipah virus infection cases and bat roost and animal sampling, 6 sites, Bangladesh, 2013–2015*

Characteristic	Outbreak site						
	1	2	3	4	5	6	All sites
Date of outbreak	2013 Jan	2013 Mar	2014 Jan	2014 Jan	2015 Jan	2015 Jan	
No. human cases	2	7	4	4	7	5	
District	Rajshah	Manikgonj	Faridpur	Magura	Faridpur	Naogaon	
Bat roost sampled, Y/N	Y	Y	Y	Y	N	Y	
Ct value, median (IQR)	38.62 (38.50–38.72)	29.94 (29.60–33.90)	37.5	N/A	N/A	37.25	
Nipah virus RNA in urine underneath roost	Pos	Pos	Pos	Neg	N/A	Pos	
Distance from households to bat roosts, m, median (IQR)	609 (367–1,200)	770 (400–1,970)	583 (499–812)	325 (152–1,040)	359 (162–520)	1,065 (224–1,190)	
Months from outbreak to sampling animals (sampling date)	9 mo (2013 Oct)	8 mo (2013 Nov)	4 mo (2014 May)	6 mo (2014 Jul)	5 mo (2015 Jun)	8 mo (2015 Sep)	
Domestic animals sampled and tested, no. (%)							
Cattle	0/43	0/47	0/50	0/50	1/45 (2)	2/39 (5)	3/274 (1)
Goat	0/49	0/51	0/50	0/50	0/69	0/61	0/330
Total	0/92	0/98	0/100	0/100	1/114 (1)	2/100 (2)	3/604 (1)
Peridomestic animals sampled and tested, no. (%)							
Dog	1/36 (3)	0/28	1/27 (4)	1/35 (3)	0/44	2/19 (11)	5/189 (3)
Cat	0/14	1/22 (5)	3/15 (20)	0/8	0/6	0/20	4/85 (5)
Rat	0/28	0/23	0/36	0/17	0/17	0/21	0/142
House shrew	0/12	0/25	0/13	0/33	0/23	0/30	0/136
Total	1/90 (1)	1/98 (1)	4/91 (4)	1/93 (1)	0/90	2/90 (2)	9/552 (2)

*Ct, cycle threshold; IQR, interquartile range; NA, data not available; neg, negative; pos, positive.

During October 2013–October 2015, at 4–9 months after onset in human case-patients, we revisited the villages surrounding each bat roost to test for evidence of infection among domestic animals (e.g., cattle and goats) and peridomestic animals (e.g., dogs, cats, rodents, and house shrews) living near the bat roosts; none of the villages had animal cases during outbreaks. Starting from the household closest to each bat roost, we approached each nearby household to determine whether they owned cattle, goats, cats, or dogs that had resided there for the previous month; households owning any of these animals were asked to participate in the study. We continued this process until we reached the target sample size of 100 domestic animals (0.1% prevalence, 3% null, 80% power, 95% CI, and design effect of 2) and 90 peridomestic animals (1.0% prevalence, 3% null, 80% power, 95% CI, and design effect of 2) in each village. In the first 2 sites we could not sample the targeted number of domestic animals because of unavailability according to our inclusion criteria. 

We asked each animal owner about rearing practices, the health status of animals during the month before the human Nipah outbreak, and whether the animal was fed date palm sap or fruit found on the ground or was ever observed to scavenge bat carcasses or bat placentas. For blood collection, we manually restrained cattle, goats, dogs, and cats and captured rats (*Rattus rattus*, *Bandicota bengalensis*, *B. indica*) and shrews (*Suncas murinus*) by using baited traps in and around the houses of the study participants. We sent serum samples to the US Centers for Disease Control and Prevention (Atlanta, GA, USA), where a human IgG ELISA (*10*) was adapted and optimized to test animal serum by using alternative positive and negative control serum and horseradish peroxidase–conjugated Pierce Recombinant Protein A/G (ThermoFisher Scientific, https://www.thermofisher.com). The ELISA was developed by infecting Vero-E6 cells with whole NiV. The study protocol was reviewed and approved by Research Review Committee and Animal Experimentation Ethics Committee of icddr,b.

We sampled 1,156 animals from 369 households at 6 locations (Table 1). No sick animals were reported by animal owners around the time of the human outbreak or during sampling. Previous studies indicate that, except for cats, NiV does not cause severe infection among the animals that we sampled (*11*). Each study site had >1 animal with evidence of IgG against NiV. Serum samples from 1% of cattle (3/274), 3% of dogs (5/189), and 5% of cats (4/85) had evidence of NiV antibodies (Table 1). Thirteen cattle (5%) and 3 goats (1%) were fed date palm sap, but none had evidence of NiV antibodies. One third of cattle (91/274) and goats (110/330) were fed dropped fruit, including 2 cattle with NiV antibodies (Table 2). No owner of either dogs or cats reported observing their animal feeding on bat carcasses or bat placentas.

**Table 2:** Domestic animals fed with dropped fruit and date palm sap by households in 6 sites where spillover human NiV infection cases were detected, Bangladesh, 2013–2015*

Parameters	Value	p value†
Domestic animals fed with dropped fruit	201 (100)	
NiV antibody–positive cattle	2/91 (2)	0.21
NiV antibody–positive goats	0/110	
Domestic animals fed with date palm sap	16 (100)	
NiV antibody–positive cattle	0/13	
NiV antibody–positive goats	0/3	

*Values are no. (%) or no. positive/no. tested (%). NiV, Nipah virus. †By χ^2^ test; p<0.05 considered significant.

## Conclusions

No animal owners reported sick animals at the time of the human Nipah outbreak. However, the long gap between the outbreak and the survey may have led to underreporting of clinical signs by owners. Pteropid bats forage on fruit trees near human residences in Bangladesh and drop partially eaten fruits on the ground (*12*). Those partially eaten fruits could act as a source of infection with NiV (or related henipaviruses) for cattle and goats (*13*). Serologic evidence of NiV infection in goats was reported from Malaysia during the human Nipah outbreak during 1998–1999 (*14*). Cattle and goats were very rarely fed date palm sap but frequently fed dropped fruit, and nearly all livestock with NiV antibodies were fed dropped fruit. A previous study from Bangladesh detected NiV antibodies among cattle, goats, and pigs using a Luminex assay (biotechne, https://www.bio-techne.com), but none of the animals with antibodies detected in that study had antibodies detected by CDC’s in-house ELISA used in our study (*5*). A conjugate that is more general to catching all species was used because controls for each of the species tested, except for pigs, were not available. CDC’s in-house ELISA was previously used to test for Hendra virus and NiV antibodies and showed cross-reaction for Hendra virus IgG (*6*). The positive animals in the earlier study could have been exposed to NiV or a related henipavirus, given that *P. medius* bats carry genetically diverse genotypes that might be antigenically similar.

Our study found that a small proportion of domestic animals (e.g., cattle and goats) had evidence of NiV antibodies, whereas evidence of antibodies were more common in peridomestic animals (e.g., cats and dogs). Because peridomestic dogs and cats roam around freely, they may have scavenged bat carcasses or placentas underneath the roost without knowledge of the owners. Previous studies have described henipavirus infection among dogs and cats in other countries (*4*,*11*,*15*). Characterizing the risk factors for infection and the potential role of domestic animals as intermediate or amplifying hosts in Bangladesh would provide a more complete understanding of the ecology of NiV in Bangladesh. In addition, to avoid potential spillover, owners of domestic animals could be advised to not feed them dropped fruit.
